# Gender differences in characteristics of physical and sexual victimization in patients with dual diagnosis: a cross-sectional study

**DOI:** 10.1186/s12888-017-1413-0

**Published:** 2017-07-25

**Authors:** Marleen M. de Waal, Jack J. M. Dekker, Martijn J. Kikkert, Maaike D. Kleinhesselink, Anna E. Goudriaan

**Affiliations:** 1 Department of Research, Arkin Mental Health Care, Klaprozenweg 111, 1033 NN, Amsterdam, The Netherlands; 2 Academic Medical Center, Department of Psychiatry, Amsterdam Institute for Addiction Research, University of Amsterdam, Amsterdam, The Netherlands; 3 Department of Clinical Psychology, Vrije Universiteit Amsterdam, Amsterdam, The Netherlands

**Keywords:** Violence, Physical abuse, Sexual assault, Victimization, Dual diagnosis, Substance use disorder, Severe mental illness

## Abstract

**Background:**

Patients with substance use disorders and co-occurring mental health disorders are vulnerable to violent victimization. However, no evidence-based interventions are available to reduce patients’ vulnerability. An exploration of the characteristics of physical and sexual violence can provide valuable information to support the development of interventions for these patients. This study aimed to examine gender differences in characteristics of violent victimization in patients with dual diagnosis.

**Methods:**

In this cross-sectional survey study recent incidents of physical and sexual assault were examined with the Safety Monitor in 243 patients with dual diagnosis. Chi-square tests were used to examine gender differences in the prevalence of physical and sexual victimization. Fisher’s exact tests and Fisher-Freeman-Halton exact tests were used to determine whether there were significant differences between victimized men and women with regard to perpetrators, locations, reporting to the police and speaking about the assault with others.

**Results:**

There was no significant difference in the prevalence of physical violence in men (35%) and women (47%) with dual diagnosis. There was a significant association between gender of the victim and type of perpetrator (*P* < .001). Men were most often physically abused by a stranger or an acquaintance, whereas women were most frequently abused by an (ex)partner. Sexual violence was more prevalent in women (29%) compared to men (4%) (*P* < .001). Patients with dual diagnosis were unlikely to report incidents of physical abuse and sexual assault to the police and to speak about it with caregivers.

**Conclusions:**

Characteristics of physical violence are different for men and women with dual diagnosis. Women with dual diagnosis are more often victims of sexual violence compared to men. Interventions aimed at reducing patients’ vulnerability for victimization should take gender differences into account.

## Background

Almost half a million people die each year as a result of interpersonal violence [1]. Millions more suffer from non-fatal violence and it’s far-reaching health consequences [1]. Alongside physical injuries, interpersonal violence can lead to depression, unemployment, drug and alcohol misuse and posttraumatic stress disorder [1, 2]. Men are more often victims of fatal as well as non-fatal physical violence compared to women. In men, in most cases the perpetrator is a stranger [3]. Domestic violence and sexual victimization are more prevalent in women. In women, in most cases the perpetrator of physical and sexual assault is an acquaintance or intimate partner [2, 3]. In the United States only 48% of physical assaults is reported to the police [4]. Victims of sexual violence are even less likely to report the abuse [4–6].

Vulnerable groups such as the elderly [2], people with disabilities [7, 8] and the homeless [9] have a higher risk of becoming a victim of abuse. The last decades, numerous studies in different countries showed that violent victimization is also more prevalent in patients with severe mental illness [10, 11] and patients with substance use disorders [12]. In these patients, victimization has consistently been associated with more severe symptomatology, homelessness, more substance use, more psychiatric hospitalizations and engagement in criminal activities [10–12]. While numerous studies and several reviews cite prevalence rates and risk factors for violent victimization in patients with mental illness, only few studies with small sample sizes address *characteristics* of the incidents. Therefore, a clear picture of the characteristics of violence in patients with mental disorders is lacking. One study reports that patients with severe mental illness are mostly victimized by family members [13], whereas two other studies report that most patients are victimized by strangers, followed by acquaintances such as neighbors and to a much lesser extent by (ex)partners and family members [14, 15]. Patients with severe mental illness seem to be most often violently victimized in their own home, whereas patients with substance disorders are most often violently victimized in public [14, 16]. Unfortunately, none of these studies report results disaggregated by gender, probably due to small sample sizes. It is thus unclear whether the gender differences that exist in the general population can be translated to patients with mental illness.

Patients with both psychiatric and substance use disorders (dual diagnosis) are even more vulnerable to victimization compared to patients with mental illness or substance use disorder only [17]. Dual diagnosis are very common in the community and treatment settings [18, 19], yet very little is known about victimization of this patient group. Caregivers need to be aware of incidents of violence to be able to provide support to patients. There is evidence that victimization is often undetected in health care facilities [20], but it is unclear whether patients are inclined to speak about incidents of victimization with caregivers, partners, family members or friends. Furthermore, it has been hypothesized that individuals with mental illness might be less likely to report victimization to the police, because of fear of not being believed or taken seriously [21]. However, there is no evidence to support this hypothesis.

There is an urgent need for evidence-based interventions to reduce the risk of victimization of patients with mental illness and thereby improve their well-being [22–24]. First attempts to develop interventions and investigate the effectiveness are underway [25, 26]. An exploration of characteristics of physical and sexual violence in patients with dual diagnosis can provide valuable information to support the development of interventions for these patients. Furthermore, it provides caregivers with more insight in a phenomenon often overlooked [20]. To the best of our knowledge, this is the first study to examine gender differences in characteristics of violent victimization in patients with dual diagnosis. Recent incidents of physical and sexual assault are explored with regard to the perpetrator, the location, reporting to the police and speaking about it with others. Consistent with studies in the general population, we hypothesize that men with dual diagnosis are most often victimized by strangers while women are most often victimized by partners or ex-partners. In both men and women we expect low rates of reporting victimization to the police.

## Methods

### Participants

This cross-sectional study utilized baseline data from a randomized controlled trial designed to determine the effectiveness of a new intervention that aims to reduce victimization in patients with dual diagnosis. Complete study details are described elsewhere [25]. The sample consisted of 243 participants. Participants were patients 18 years of age or older diagnosed with substance dependence or substance abuse (involving alcohol and/or drugs) according to the *Diagnostic and Statistical Manual of Mental Disorders-IV* (DSM-IV) criteria and at least one other mental disorder on DSM-IV Axis I or Axis II. Patients could not participate in the study if they did not have sufficient understanding of the Dutch language, were not willing to provide informed consent or were not eligible for group therapy according to their case manager, due to for instance severe psychotic symptoms or anti-social behavior.

### Procedures

Patients were recruited in an addiction-psychiatry clinic and allied addiction-psychiatry outpatient care facility. Patients who met the inclusion criteria were informed and invited by a caregiver and researcher. Once the procedures had been fully explained and patients had given written informed consent, a face-to-face assessment was performed. The assessments were conducted at the treatment facility by a researcher (master’s degree) and trained master students (with a bachelor’s degree) in clinical psychology. The inclusion period lasted two years, from April 2014 to April 2016.

### Measures

#### Demographics

Demographic characteristics were collected during the assessment. Current DSM-IV diagnoses were determined by psychiatrists and extracted from the electronic patient record.

#### Victimization

Victimization was measured with the Safety Monitor (in Dutch: Veiligheidsmonitor), developed by the Dutch Ministry of Security and Justice [27]. The Safety Monitor is an adequate self-report instrument used by the governmental institution Statistics Netherlands to measure victimization on a large scale. It strongly resembles the International Crime Victimization Survey (ICVS) [28]. The Safety Monitor was used to examine whether participants experienced different types of crime in the last 12 months. Each crime reported is followed by an exploration of the most recent incident. Data regarding physical abuse and sexual assault were used in this study. The Safety Monitor contains questions regarding the perpetrator and the location of the abuse. Perpetrators can be strangers, (ex)partners, relatives, neighbors, fellow patients or acquaintances. The category ‘acquaintance’ includes: friends of friends, friends or relatives of ex-partners and drug dealers. Furthermore, it is examined whether the incident was reported to the police and whether participants spoke about the incident with others. If relevant, reasons for not reporting to the police were examined. To be able to exemplify our findings we extended the Safety Monitor with an open question which invited participants to shortly describe what led to the incident.

### Data analysis

Statistical analyses were performed in SPSS Statistics 22.0. Chi-square tests and independent t-tests were used to examine gender differences in clinical and demographic characteristics and prevalence of physical and sexual victimization. Patients that were a victim of physical and/or sexual violence were included in the subsequent analyses. Fisher’s exact tests and Fisher-Freeman-Halton exact tests were used to determine whether there were significant differences between victimized men and women with dual diagnosis with regard to perpetrators, locations, reporting to the police and speaking with others. The Fisher-Freeman-Halton exact test is an extension of the Fisher’s exact test that can be applied to unordered r x c Tables. A Fisher-Freeman-Halton exact test was used to determine whether there is an association between type of perpetrator and location of violence in the four most frequently reported perpetrators. Statistical significance was set at *P* < .05.

## Results

In total, 243 participants were included in the study. During the inclusion period, there were 487 eligible patients, of which 216 (44%) declined to participate, 18 (4%) did not show up on appointments and 10 (2%) withdrew from participations during the first assessment. The most frequently reported reasons to decline were being unmotivated for treatment (*N* = 65) and not having time due to work or other therapy programs (*N* = 49). On average, the 243 included participants were 42.3 years old (SD = 10.9) and were diagnosed with 3.7 mental health disorders according to DSM-IV (SD = 1.4). The majority was male (70.4%).

### Demographic and clinical characteristics by gender

Table 1 lists demographic and clinical characteristics of the 243 participants disaggregated by gender. Personality disorders were more prevalent in women compared to men (61% vs. 27%, *χ* ^*2*^ = 25.428, *df* = 1, *P* < .001). There were no other significant gender differences in demographic and clinical characteristics.

**Table 1:** Demographic and clinical characteristics and violent victimization of men and women with dual diagnosis

	Patients with dual diagnosis (*N* = 243)			
	Men (*N* = 171)	Women (*N* = 72)	t/χ^2^	*P*
Age [M (SD)]	41.8 (11.0)	43.4 (10.4)	1.100	.273
Unemployed [%]	91.8	95.8	1.259	0.262
Substance use disorders [%]^a^				
Alcohol	63.2	63.9	.012	.914
Cannabis	49.7	37.5	3.039	.081
Cocaine	44.4	43.1	.040	.842
Opioid	21.1	27.8	1.292	.256
Sedatives	17.0	27.8	3.684	.055
Other substances	12.9	15.3	.251	.616
Psychiatric disorders Axis I [%]^a^				
Psychotic disorder	42.1	29.2	3.590	.058
Mood disorders	22.2	22.2	.000	1.000
Anxiety disorders	21.6	20.8	.019	.889
Attention-deficit/hyperactivity disorder	8.8	6.9	.224	.636
Other disorder	13.5	6.9	2.104	.147
Psychiatric disorder Axis II [%]^a^				
Personality disorder	26.9	61.1	25.428	**<.001**
Intellectual disability	12.9	12.5	.006	.938
Total number of disorders [M (SD)]	3.7 (1.4)	3.8 (1.3)	−.831	.407
Violent victimization [%]				
Physical violence	34.5	47.2	3.470	.063
Sexual violence	4.1	29.2	31.243	**<.001**

^a^Disorders can co-occur

^b^Significant findings are shown in bold

### Violent victimization

Of the 171 male participants 59 (35%) reported physical violence in the last 12 months and 7 (4%) reported sexual violence. Of the 72 female participants 34 (47%) reported physical violence in the last 12 months and 21 (29%) reported sexual violence. There was no significant gender difference in the prevalence of physical violence. Sexual violence was significantly more prevalent in women compared to men (*χ* ^*2*^ = 31.243, *df* = 1, *P* < .001, Table 1). Participants that were a victim of physical and/or sexual violence (*N* = 102) were included in the subsequent analyses.

### Physical violence

In victims of physical violence (*N* = 93) there was a significant association between gender of the victim and type of perpetrator (*P* < .001), as shown in Table 2. Male patients with dual diagnosis were most often physically victimized by a stranger (35.6%) or an acquaintance (32.2%), followed by a relative (11.9%), (ex)partner (8.5%), fellow patient (8.5%) and neighbor (3.4%). Female patients were most often victimized by an (ex)partner (41.2%), followed by a stranger (26.5%), fellow patient (11.8%), acquaintance (11.8%) and neighbor (9.1%). Furthermore, there was a significant association between gender of the victim and location where the physical violence took place (*P* = .007). Male patients with dual diagnosis were most often physically victimized in public (55.9%), followed by at home (37.3%) and in another person’s home (6.8%). Female patients were most often victimized at home (44.1%) followed by in public (32.4%), in a care facility (14.7%) and in another person’s home (8.8%).

**Table 2:** Characteristics of most recent incident of physical victimization in men and women with dual diagnosis

	Total (*N* = 93)	Men (*N* = 59)	Women (*N* = 34)	
Perpetrator	%	%	%	*P*
Stranger	32.3	35.6	26.5	**<.001** ^a^
(Ex)partner	20.4	8.5	41.2	
Relative	7.5	11.9	0.0	
Neighbor	5.4	3.4	9.1	
Fellow patient	9.7	8.5	11.8	
Acquaintance	24.7	32.2	11.8	
Location	%	%	%	*P*
At home	39.8	37.3	44.1	**.007** ^a^
Other’s home	7.5	6.8	8.8	
In public	47.3	55.9	32.4	
Clinic/daycare	5.4	0.0	14.7	
Reported to police	%	%	%	*P*
Yes	16.1	8.5	29.4	**.017** ^b^
No	83.9	91.5	70.6	
Speaking with others	%	%	%	*P*
Yes	86.0	84.7	88.2	.762^b^
No	14.0	15.3	11.8	

^a^Fisher-Freeman Halton test

^b^Fisher’s exact test

^c^Significant findings are shown in bold

Men with dual diagnosis were significantly less likely to report physical victimization to the police compared to women (8.5% vs. 29.4%, *P* = .017, Table 2). In the group of participants that did not report the physical abuse to the police, the most cited reasons for not reporting were: belief that the police could or would not do anything to help (26.9%), fear of reprisal or getting the offender in trouble (25.6%), dealt with it in another way (16.7%) and fear to get in trouble with the law themselves (15.4%). There was no significant gender difference in speaking with others about the incident. Most of the victims of physical abuse spoke about the abuse with at least one other person (86.0%). The most cited persons with whom victims spoke about the abuse were: a friend (41.9%), relative (30.1%), caregiver (29.0%) and partner (15.1%).

### Sexual violence

One of the 7 male patients that reported sexual violence refused to answer questions about characteristics of the incident. The remaining number of males who reported sexual assault (*N* = 6) was too low to make a reliable comparison with the female sexual assault victims (*N* = 21). Therefore we will describe the characteristics of the total group of patients with dual diagnosis that were a victim of sexual assault. As shown in Table 3, most victims reported sexual assault by a stranger (40.7%) followed by an (ex)partner (18.5%) and fellow patient (18.5%). Patients were most often sexually victimized at home (37.0%) followed by in another person’s home (29.6%), in public (18.5) and in a care facility (14.8%).

**Table 3:** Characteristics of most recent incident of sexual victimization in men and women with dual diagnosis

	Total (*N* = 27)	Men (*N* = 6)	Women (*N* = 21)
Perpetrator	%	%	%
Stranger	40.7	83.3	28.6
(Ex)partner	18.5	0.0	23.8
Relative	0.0	0.0	0.0
Neighbor	7.4	0.0	9.5
Fellow patient	18.5	16.7	19.0
Acquaintance	14.8	0.0	19.0
Location	%	%	%
At home	37.0	0.0	47.6
Other’s home	29.6	50.0	23.8
In public	18.5	50.0	9.5
Clinic/daycare	14.8	0.0	19.0
Reported to police	%	%	%
Yes	18.5	16.7	19.0
No	85.2	83.3	81.0
Speaking with others	%	%	%
Yes	74.4	67.7	76.2
No	25.6	33.3	23.8

Only 18.5% of the victims reported the sexual assault to the police. The most cited reasons for not reporting the incident were: the belief that the police could or would not do anything to help (27.3%) and fear of reprisal or getting the offender in trouble (18.2%). Most of the victims of physical abuse spoke about the abuse with at least one other person (74.4%). The most cited persons with whom victims spoke about the abuse were: a friend (37.0%), caregiver (37.0%), relative (14.8%) and partner (11.1%).

### Further exploration per type of perpetrator

We further explored the four most frequently reported perpetrators of all incidents of physical and sexual violence. As shown in Table 4, there was a significant association between the type of perpetrator and the location where the victimization took place (*P* < .001). When patients with dual diagnosis were victimized by a stranger, this mostly took place in public (71%). When patients with dual diagnosis were victimized by an (ex)partner, this mostly took place at home (71%). When patients were victimized by a fellow patient, this mostly took place at home (36%) or in a care facility (36%). Table 5 provides several incident descriptions of participants to exemplify the types of violence suffered by different perpetrators.

**Table 4:** Association between type of perpetrators and locations of violent victimization of patients with dual diagnosis

	Stranger (*N* = 41)	(Ex)partner (*N* = 24)	Fellow patient (*N* = 12)	Acquaintance (*N* = 27)	
Location	%	%	%	%	*P*
At home	12.2	70.8	35.7	25.9	**<.001** ^a^
Other’s home	12.2	8.3	14.3	22.2	
In public	70.7	12.5	14.3	51.9	
Clinic/daycare	4.9	8.3	35.7	0.0	

^a^Fisher-Freeman Halton test

^b^Significant findings are shown in bold

**Table 5:** Examples of incident descriptions of victimization of dual diagnosis patients ordered by type of perpetrator

Stranger
A stranger that I met on the streets promised me drugs, so I went with him to his place. When we were there he suddenly started to undress me.
Two guys pulled me from my bike, knocked me down and threatened me with a knife. They took the money from my wallet.
(Ex)partner
I used all our money to buy cocaine. When my partner found out she got angry and hit me.
I got home late, which made my husband think I cheated on him and therefore he beat me up.
Fellow patient
He is very short tempered and outside he suddenly kicked me in the shins because of an argument we had earlier at daycare.
We were both admitted in the clinic. He made sexually offensive remarks and touched my intimate body parts.

## Discussion

In the present study, patients with dual diagnosis were most often physically victimized by a stranger (32.3%), followed by an acquaintance (24.7%) and (ex)partner (20.4%). This is in accordance with findings in patients with severe mental illness from the Netherlands [14] and from Sweden [15], but in contrast to a study conducted in Greece [13] in which patients with severe mental illness were mostly victimized by family members (45.8%). This difference is probably due to the fact that the Greek study was conducted in an isolated, rural area where family members are more dependent on each other, whereas the present study is conducted in the capital city of the Netherlands. In our study, physical abuse of patients with dual diagnosis mostly took place in public (47.3%), or in a patient’s own home (39.8%). Previous studies imply that patients with substance use disorders are mostly victimized in public, whereas patients with severe mental illness are more often victimized at home [14, 16]. Patients with co-occurring mental health and substance use disorders seem to be vulnerable to physical abuse in both locations. Perpetrators of sexual assault were mostly strangers (40.7%) followed by (ex)partners (18.5%). Sexual violence mostly took place at home (37.0%) followed by in another’s home (29.6%) and in public (18.5%). This is largely in line with findings in patients with severe mental illness from Sweden [15].

In contrast to previous studies [13–15], we examined gender differences with regard to the perpetrators and locations of physical violence. We found that men were mostly physically abused in public, whereas women were most often physically abused at home. In men, in most cases, the perpetrator was a stranger, whereas in women in most cases the perpetrator was an (ex)partner. This is in accordance with our hypothesis and with findings in the general population [2, 3]. We found that sexual assault was more prevalent in women with dual diagnosis compared to men. Too few men reported sexual assault in the 12 months prior to taking the assessment to draw reliable conclusions on the effect of gender on characteristics of sexual victimization. Women were mostly sexually assaulted by a stranger. This is in contrast to the general population, in which 62% of women who are raped during adulthood are raped by an intimate partner [3].

Consistent with our hypothesis we found that patients with dual diagnosis were unlikely to report physical victimization to the police (16.1%). Men were even more unlikely to report physical abuse to the police compared to women. We found lower rates of reporting in patients with dual diagnosis compared to the general population of the Netherlands in which it is estimated that 44% of incidents of violent victimization are reported to the police [29]. Interestingly, the gender difference that we found in patients with dual diagnosis does not exist in the general population [5, 6, 29]. Women with dual diagnosis reported physical victimization in 29.4% of the cases and sexual victimization in 19.0% of the cases. Whilst these women are less inclined to speak to the police compared to women from the general population, the pattern is similar: sexual violence is less likely to be reported than physical violence [4–6]. For both physical and sexual assault, the most cited reasons for not reporting the incident to the police were the belief that the police could or would not do anything to help and fear of reprisal or getting the offender in trouble. Furthermore, with regard to physical abuse, victims reported that they dealt with the incident in another way or did not report the incident because of fear to get in trouble with the law themselves. Similar beliefs and fears also exist in the general population, except for two differences [4, 5]. First, the percentage of people who believe the police could or would not do anything to help is larger in patients with dual diagnosis compared to the general population. Second, patients report more fear to get in trouble with the law themselves. This might (partly) explain why rates of reporting are lower in patients with dual diagnosis. The ‘stratification hypothesis’ [30] states that social-economic disadvantaged people are more inclined to try to deal with problems themselves without involving the justice system. The relationship between socioeconomic status and victim reporting may be mediated by the factor trust in (the effectiveness of) the police [31]. A subculture of people who are somewhat alienated from society may develop alternative ways of dealing with crime, in which people are seen as responsible for their own safety and in which involving the police is seen as weak and cowardly [31, 32]. This theory could be applicable to patients with dual diagnosis, who have to engage themselves in the illegal circuit to obtain drugs and who, on average, are more inclined to engage in criminal behavior themselves [33]. The concern that their own illegal activities might come up may detain patients from making contact with the police [32]. Finally, the lower tendency of patients with dual diagnosis to report victimization to the police may be explained by the finding that in general, victims who were using drugs or drinking alcohol when they were victimized are less likely to notify the police [5]. Future studies should address these hypotheses.

Most victims of physical abuse and sexual assault spoke about the incident with at least one other person (respectively 86.0% and 74.4%). Victims mostly spoke with friends and to a lesser extend with caregivers and relatives. In the general population, many victims who do not report to the police use alternative help-seeking strategies such as seeking support from friends, family or social services [6]. This can provide social support and comfort to crime victims. The availability and benefits of support from friends, family and caregivers need to be further explored in patients with dual diagnosis. Future studies should examine whether seeking help from others and reporting to the police influence well-being and risk of future victimization and how patients’ willingness to seek help can be raised.

The most important strength of this study is the amount of detailed information collected in a sample of patients with dual diagnosis. However, the study is not without limitations. First, the experiences of physical and sexual violence were examined with a self-report questionnaire, which is subject to memory bias. Second, we only have information about the most recent incidents of physical and sexual violence and therefore cannot draw conclusions on perpetrators, locations and police reporting in patients who are victims of multiple incidents of violence. Finally, it was impossible to draw conclusions on gender differences with regard to sexual violence, since a small number of men experienced sexual violence. This limitation also applies to studies in the general population [34].

## Conclusions and implications

There are similarities as well as differences between men and women with dual diagnosis in characteristics of physical and sexual victimization. Both men and women are frequently violently victimized in public by strangers, which suggests it may be useful for patients with dual diagnosis to gain more ‘street skills’ by learning about changeable factors that contribute to their safety in public [35, 36]. Furthermore, both men and women are frequently violently victimized by someone they know, which suggests it may be helpful for patients with dual diagnosis to gain more social skills and conflict resolution skills [25]. Women with dual diagnosis are more often a victim of sexual violence and partner violence compared to men. Recently, two new interventions have been developed that aim to reduce patients’ vulnerability for victimization [25, 26], however, these interventions do not take gender differences into account. Our findings suggest that it is necessary to address at least some of the needs of women with dual diagnosis separately. Since, to the best of our knowledge, we are the first to examine gender differences in characteristics of physical and sexual violence in patients with dual diagnosis, replication of our findings is necessary, also in other countries. Besides, further research is needed to determine differences in characteristics of victimization in different patient groups (e.g. patients with psychotic disorders, patients with personality disorders and patients with substance use disorders) and different living situations (e.g. rural vs. urban). This type of research can provide a clearer understanding of violent victimization in patient populations and result in suggestions for the further development of personalized interventions to reduce victimization in the diverse population of patients with psychiatric disorders.

## Abbreviations

DSM-IV: Diagnostic and Statistical Manual of Mental Disorders-IV
ICVS: International Crime Victimization Survey
